# Improved Gas Hydrate Kinetic Inhibition for 5-Methyl-3-vinyl-2-oxazolidinone Copolymers and Synergists

**DOI:** 10.1021/acsomega.3c03986

**Published:** 2023-07-25

**Authors:** Malcolm A. Kelland, Erik G. Dirdal, Radhakanta Ghosh, Hiroharu Ajiro

**Affiliations:** † Department of Chemistry, Bioscience and Environmental Engineering, Faculty of Science and Technology, University of Stavanger, N-4036 Stavanger, Norway; ‡ Division of Material Science, Graduate School of Science and Technology, Nara Institute of Science and Technology, 8916-5 Takayama-cho, Ikoma, Nara 630-0192, Japan

## Abstract

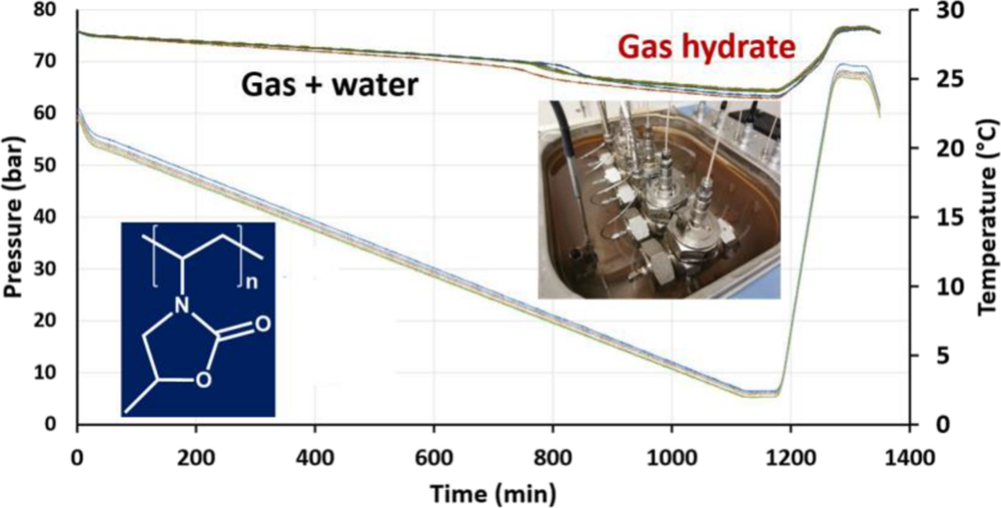

Kinetic hydrate inhibitors (KHIs) are used to prevent deposits and plugging of oil and gas production flow lines by gas hydrates. The key ingredient in a KHI formulation is a water-soluble amphiphilic polymer. Recently, polymers of a new commercially available 5-ring vinylic monomer 5-methyl-3-vinyl-2-oxazolidinone (VMOX) were investigated as KHIs and shown to perform better than some commercial KHI polymers such as poly(*N*-vinyl pyrrolidone). This initial study using slow constant cooling (SCC) in rocking cells with a synthetic natural gas has now been expanded to further explore low molecular weight PVMOX homopolymers and VMOX copolymers as well as blends with nonpolymeric synergists. A PVMOX homopolymer with improved KHI performance was found using 3-mercaptoacetic acid as a chain transfer agent in the radical polymerization of VMOX. Among a range of copolymers, VMOX:*n*-butyl acrylate copolymers in particular gave good KHI performance, better than the PVMOX homopolymer. Among the potential synergists, trialkylamine oxides (alkyl = *n*-butyl or iso-pentyl) and tetra(*n*-pentyl)ammonium bromide to 2500 ppm were found to be antagonistic with PVMOX at the test concentrations while some alcohols and glycols were synergetic. The best synergist was 2,4,7,9-tetramethyl-5-decyne-4,7-diol (TMDD). For example, a mixture of 2500 ppm TMDD with 2500 ppm PVMOX (*M*_w_ 2400 g/mol) performed significantly better than 5000 ppm PVMOX. Addition of 1250 ppm TMDD to 2500 ppm VMOX:*n*-butyl acrylate 6:4 copolymer lowered the hydrate onset temperature in SCC tests by a further 3 °C compared to the copolymer alone giving hydrate onset at 4.2 °C.

## Introduction

Gas hydrate formation in subsea gas and multiphase production flow lines is a serious problem unless treated; otherwise, it can lead to plugging of the lines.^1−5^ Kinetic hydrate inhibitors (KHIs) are a chemical method used to prevent hydrate blockages. KHI formulations include at least one water-soluble polymer plus synergists and solvents which can also act as synergists.^6−17^ Many industrial KHI polymers are based on the monomers *N*-vinylcaprolactam (VCap), *N*-vinylpyrrolidone (VP), and *N*-isopropylmethacrylamide (NIPMAm).^18^ A distribution of low molecular weights and amphiphilic side-groups with good hydrogen bonding capabilities appear to be key features of these KHI polymers.

**Figure 1:**
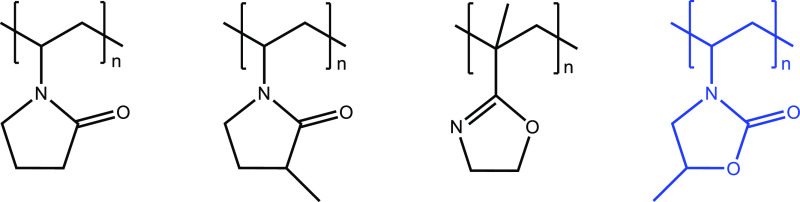
Left to right; *N*-vinyl pyrrolidone (VP), 3-methyl-*N*-vinyl pyrrolidone (3-MVP), 2-isopropenyl-2-oxazoline (iPOx), and 5-methyl-3-vinyl-2-oxazolidinone (VMOX, in blue).

Besides, VP-based polymers, several other 5-ring vinylic monomers have also successfully been used to make KHI polymers. These include isopropenyl-2-oxazoline (iPOx) and alkylated derivatives of VP such as 3-methyl-*N*-vinyl pyrrolidone (Figure 1). The monomer 5-methyl-3-vinyl-2-oxazolidinone (VMOX) only became available in multi-ton quantities recently and has already found several industrial applications.^19,20^ The VMOX structure resembles the VP monomer with a 5-membered heterocyclic ring and good hydrogen-bonding capability.

Recently, we carried out the first investigation of VMOX-based polymers as KHIs.^21^ The cloud point (*T*_cl_) of the homopolymer PVMOX at 2500 ppm in water varied from 45 to 73 °C as the molecular weight (*M*_n_) decreased from 5400 to 2400 g/mol, making the low Mn polymers. The KHI performance using a synthetic natural gas mixture was found to be between those of PVP and PVCap for similar molecular weights. In addition, no performance advantage was found using a 1:1 VMOX: VCap copolymer (*M*_w_ 5300 g/mol). Copolymerization of NIPMAm with VMOX led only to homopolymers PVMOX. As this was the very first study on VMOX-based polymers as KHIs, we wanted to explore this class in more detail using other common comonomers that might give copolymers with better KHI performance, as well as possible synergist solvents for the VMOX homo- and copolymers. Indeed, improvements were made on both fronts and we present these results here.

## Experimental Section

### Materials

*n*-Butyl acrylate (BuA, Acros, 99%), *N*-vinyl-2-pyrrolidone (VP, Merck, 99%), bisazoisobutyronitrile (AIBN, VWR, >97%), 3-mercaptopropionic acid (3-MPA, Merck, >98%), isobutyl glycol ether (iBGE, TCI, >98%), dipropylene glycol butyl ether isomer mixture (DPnB, VWR, >98%), 4-methyl-1 pentanol (Merck, 97%), 2,4,7,9-tetramethyl-5-decyne-4,7-diol (TMDD, Merck, 98%), tetrapentylammonium bromide (TPAB, Merck, >99%), and 2-propanol (iPrOH, VWR, >99%) were used as received. Tetrahydrofurfuryl methacrylate (THFMA, 97%) was supplied by Evonik, Germany. A sample of 5-methyl-3-vinyl-2-oxazolidinone monomer (VMOX, >98%) as well as PVCap homopolymer (*M*_n_ 2600 g/mol, 41.1 wt % in MEG) and *N*-vinyl-2-pyrrolidone:VCap 1:1 copolymer (VP:VCap 1:1) (*M*_n_ 2000–4000 g/mol, 53.8 wt % in water) were kindly supplied by BASF, Germany. Tri-*n*-butylamine oxide (TBAO) and triisopentylamine oxide (TiPeAO) were synthesized as previously reported.^22^ Methacryloylpyrrolidine (MAPYD) was made by the literature method.^23^

### Synthesis of PVMOX Homopolymers and Copolymers

**Figure 2:**
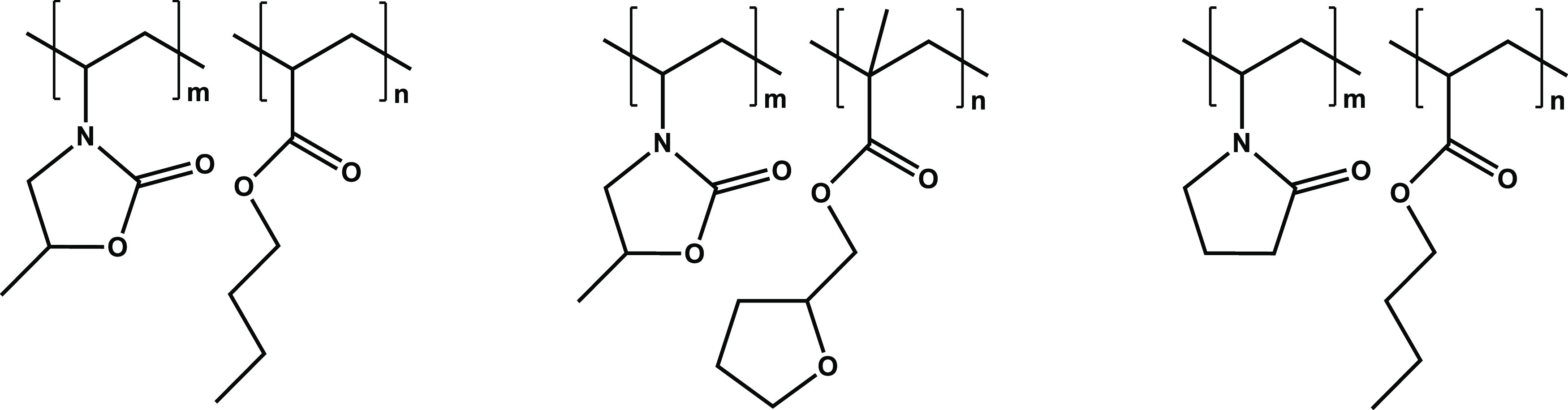
Structures of synthesized copolymers. Left to right, VMOX:BuA, VMOX:THFMA, and VP:BuA.

Both homopolymers and copolymers of PVMOX were synthesized in the same general manner. A typical example is given here for a PVMOX homopolymer: VMOX (5 g, 39.32 mmol) was dissolved in 2-propanol (10 g) in a N2-purged Schlenk flask with a bar magnet. AIBN (1.0 wt %, 0.05 g) was added carefully, and the solution was flushed three times with N_2_ using the standard pump-fill technique. The solution was then heated to 80 °C with continuous stirring and left to react under the protection of nitrogen overnight. PVMOX homopolymer came out of the solution and the remaining solvent was discarded. The moist solid was dried in vacuo on a rotary evaporator, leaving solid white PVMOX-5.4k (*M*_w_ = 5400 g/mol). The other two PVMOX homopolymers were synthesized following the same procedure except 3-MPA, which was also used to make lower molecular weight polymers. PVMOX-2.4k and PVMOX-3.1k were synthesized in the presence of 4.5 and 9 mmol 3-mercaptopropionic acid, respectively. The VMOX:BuA and VP:BuA copolymers were synthesized in 2-propanol using 9:1, 8:2, 7:3, 6:4, and 5:5 comonomer ratios in the presence of AIBN (1 w%) and 3-mercaptopropionic acid (3-MPA, 4.5 mmol). VMOX:THFMA copolymers were made in the same way but without the use of 3-MPA (Figure 2). All copolymers were left in the iPrOH solution as 25–30 wt % solutions. ^1^H NMR spectroscopy indicated full conversion based on the lack of vinylic protons.^21^ GPC molecular weight analysis was carried out in DMF as a solvent at 40 °C, using superH3000 and GMH columns of Tosoh company, and PSS standards.

### Cloud Point (*T*_cl_) Measurement

*T*_cl_ was determined by dissolving the polymer in deionized water to 2500 ppm, followed by fairly rapid heating (<10 °C/min). The temperature at which clouding of the solution was first observed was taken as the cloud point (*T*_cl_). The solution was cooled below the *T*_cl_ and when completely clear it was reheated, this time at a slower heating rate (2–3 °C/min), and the *T*_cl_ was recorded again. This was repeated twice at the slower heating rate to check reproducibility, and this was taken as the final *T*_cl_ value.

### KHI Performance Tests

**Figure 3:**
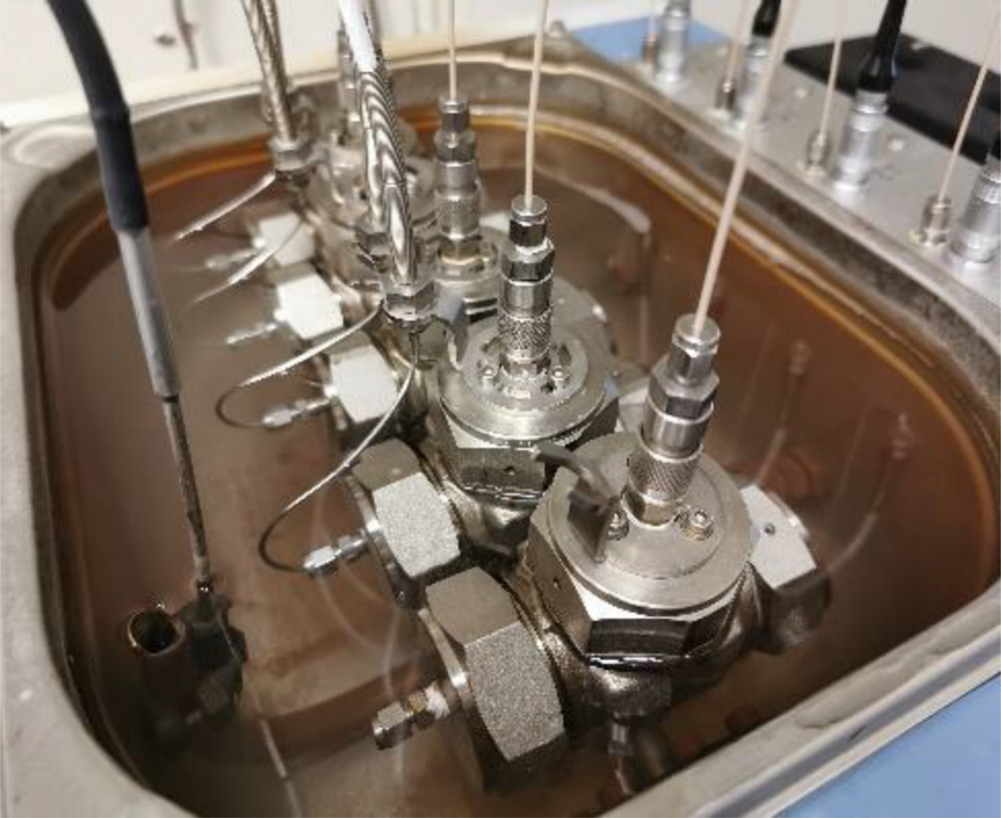
Five high-pressure steel cells in the temperature-controlled rocker rig.

All KHI performance experiments were carried out in a parallel series of five high-pressure rocking cells placed inside a temperature-controlled water bath. The rig was supplied by PSL Systemtechnik, Germany (Figure 3).^21,22^ A synthetic natural gas (SNG) blend was used (Table 1) made by Yara Praxair, Norway. The composition was analyzed to be within ±0.1% of all the required concentrations. The equilibrium temperature (*T*eq) for sII gas hydrate at 76 bar of SNG was predicted to be 20.5 °C by PVTSim software, Calsep.

**Table 1:** Composition of the Synthetic Natural Gas (SNG) Mixture

component	mol %
nitrogen	0.11
*n*-butane	0.72
isobutane	1.65
propane	5.00
CO_2_	1.82
ethane	10.3
methane	80.4

The slow constant cooling (SCC) test method was used to evaluate the KHI performance of all polymers and blends with potential synergists. The test method has been used by our group for many years using the same equipment and SNG, enabling us to compare the performance of new KHIs to a range of previously tested KHIs, in particular the VMOX polymers from the first study.^21^ A summary of the SCC test method used is as follows:

1. The test polymer was dissolved in 105 mL of deionized water. Preparation was done 24 h prior to the KHI test. 20 mL of this test solution was added to each cell.
2. Each cell was purged with SNG and then vacuum was applied to remove the air in the system. This was then repeated.
3. Approximately 76 bars of SNG were loaded to each cell at 20.5 °C and each cell shut individually at the gas inlet/outlet valves.
4. The cells were rocked and slowly cooled at a rate of 1 °C/h. Pressure and temperature data were recorded by sensors.

**Figure 4:**
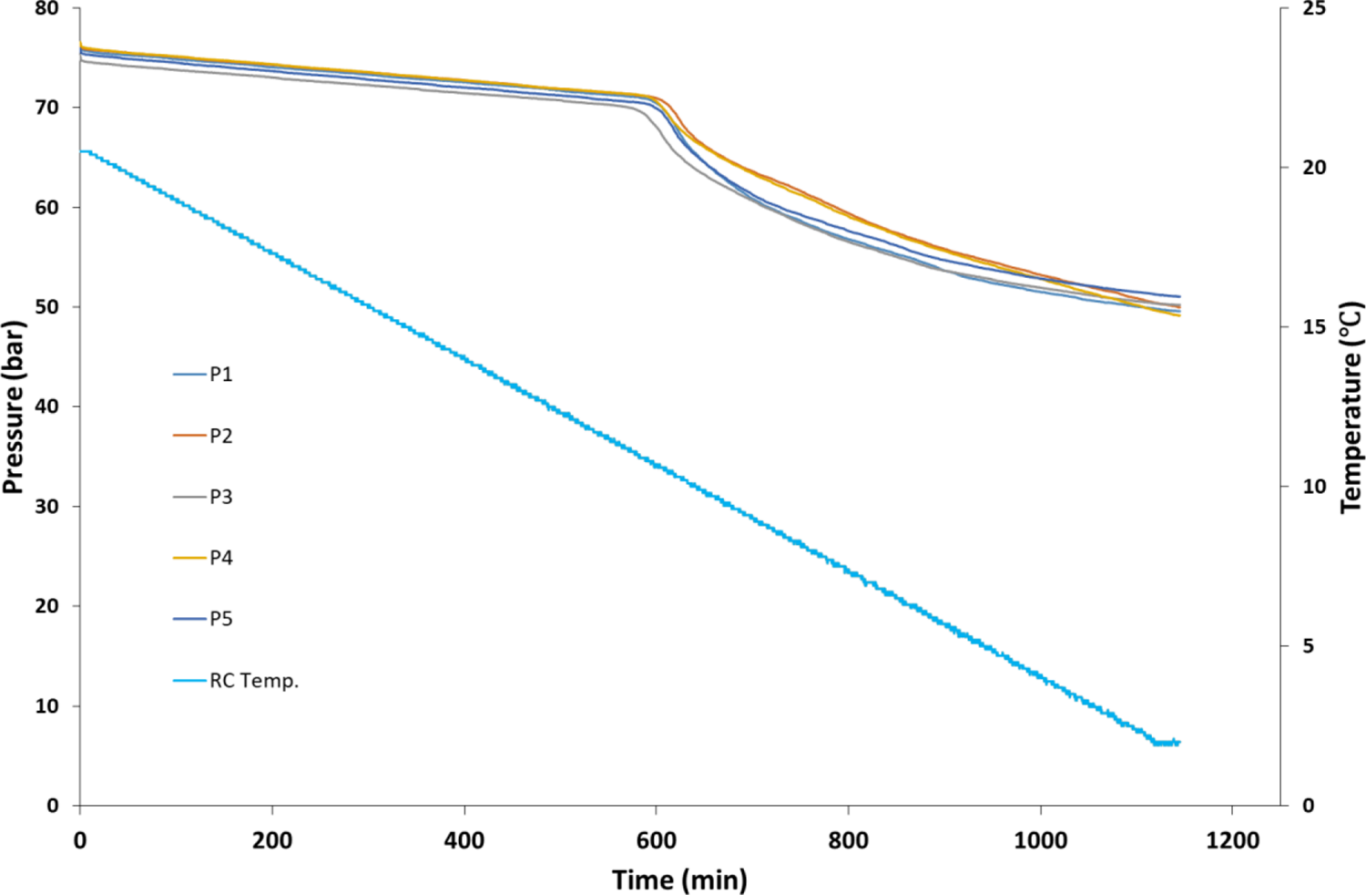
Pressure and temperature vs time curves obtained for a set of five SCC tests.

An example of the data obtained (pressure and temperature versus time) from a set of five separate parallel rocking cells on the same polymer is shown in Figure 4.

**Figure 5:**
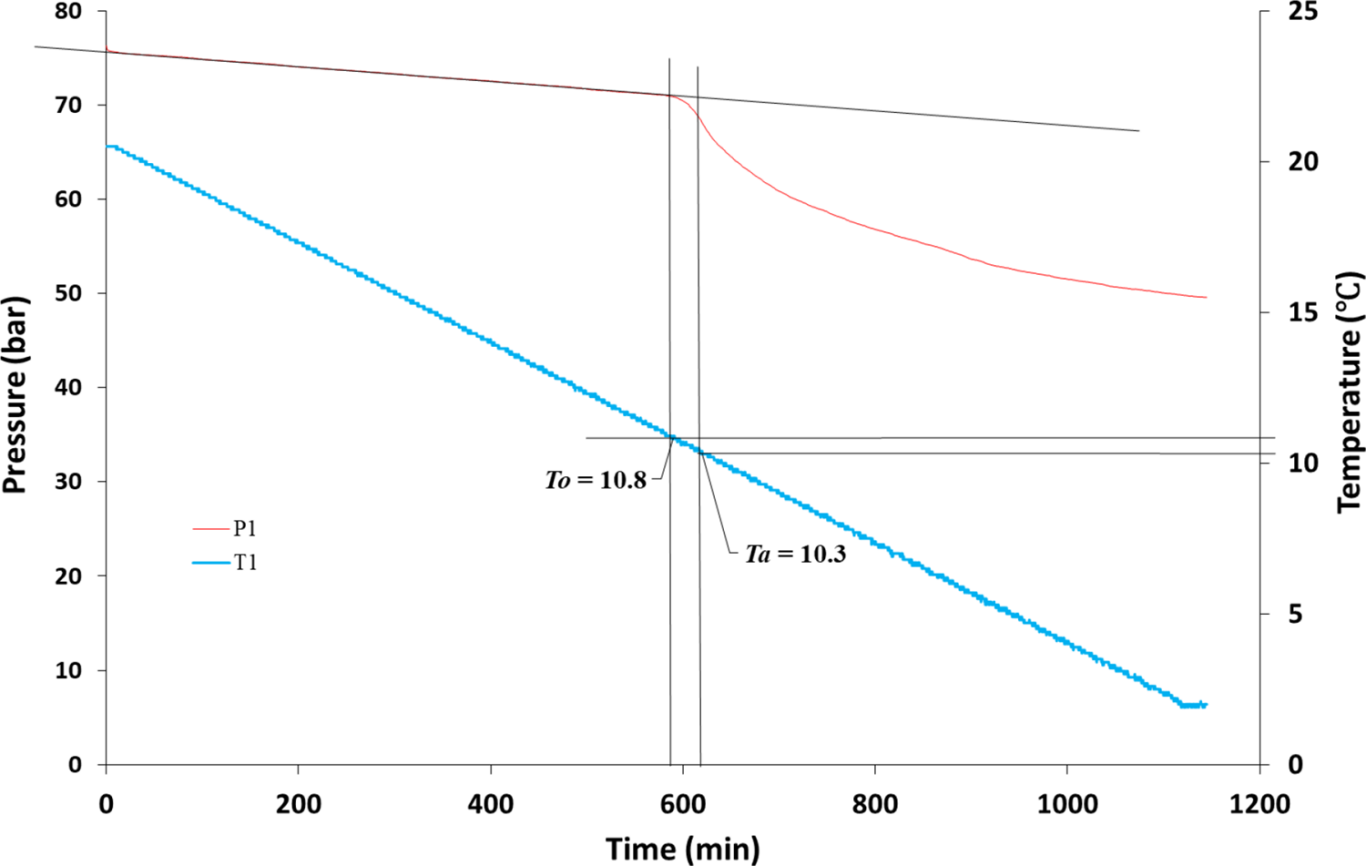
Determination of *T*_o_ and *T*_a_ values from the pressure/temperature data vs time for an SCC screening test.

Two parameters were determined from the data obtained, the hydrate onset temperature (*T*_o_) and rapid hydrate formation temperature (*T*_a_) (Figure 5). In the closed system, the pressure decreased linearly due to the constant temperature decrease. Once gas hydrates begin to form, the pressure deviates from the original linear track. The corresponding temperature at this first pressure drop is *T*_o_. The temperature at the first fastest pressure drop point is called *T*_a_. In the example in Figure 5, *T*_o_ and *T*_a_ for this cell were found to be 10.8 and 10.3 °C, respectively. Generally, 5–10 individual experiments were carried out for each polymer sample. For a set of 5–10 experiments, we typically observe a 10–15% margin of error in *T*_o_ and *T*_a_ values which was also the case in this study. This is due to the stochastic nature of the hydrate nucleation process. No bias was observed between any of the five cells, such as one cell regularly giving higher or lower *T*_o_ and *T*_a_ values than the other four. The *T*_o_ value is a more important parameter than *T*_a_ for determining the KHI performance since operators preferably want to completely stop macroscopic hydrate formation in flow lines. However, a measure of the ability to stop hydrate crystal growth at a given subcooling can be found from the *T*_o_ – *T*_a_ value.

To evaluate if there is a significant difference between sets of *T*_o_ values for two polymers, we carried out statistical *t*-tests and determined the *p*-value. The *t*-test is a well-known statistical method to evaluate if there is a significant difference between two sets of data, which in our case can help rank the KHIs.^24^ A *p*-value is calculated, usually by software (e.g., add-on in Excel), and if the *p*-value is less than 0.05, it is considered that there is a significant difference between the two sets of data at the 95% confidence level. Thus, a *p*-value of less than 0.05, between two sets of *T*_o_ values, indicates a 95% confidence that the performance of one KHI is better than another.

## Results and Discussion

### PVMOX Homopolymer Synthesis and KHI Performance

The KHI test results for all new VMOX-based polymers using the SCC method are summarized in Table 2. PVMOX had been shown previously to give better performance and higher cloud point when the molecular weight (weight average, *M*_w_) was decreased from 5400 to 2400 g/mol. The latter polymer was made using a chain transfer agent, 3-MPA. We wanted to reduce the molecular weight value even further, so we made PVMOX with a greater amount of 3-MPA. This time the *M*_w_ was found to be 3100 g/mol, so this did not help lower the *M*_w_. The *T*_cl_ in DI water was found to be 58 °C, which fitted a trend of decreasing *T*_cl_ with increasing *M*_w_. Interestingly, this polymer PVMOX-3.1k gave a significant and slightly better KHI performance than polymers with higher and lower *M*_w_ values, PVMOX-5.4k and PVMOX-2.4k (Table 2). The *p*-value between the new polymer and the old polymers was less than 0.05 from a statistical *t*-test analysis.^24^

**Table 2:** Summary of SCC Rocking Cell Tests with SNG and 2500 ppm Polymer Solution

polymer	*M*_w_, g/mol (PDI)	*T*_cl_, °C	Av. *T*_o_, °C	Av. *T*_a_, °C	*T*_o_ – *T*_a_, °C
no additive			17.9	17.0	0.7
PVCap	2600 (1.81)	33	10.1	9.6	0.5
PVP	3400 (1.1)	>100	11.3	10.5	0.8
poly(VP:VCap) 1:1	2000–4000	85	8.9	6.8	2.1
poly(VP:BuA) 9:1	bimodal 210–5000	>100	12.4	11.2	1.2
poly(VP:BuA) 8:2	bimodal 280–5000	40	8.1	7.9	0.2
PVMOX-5.4k	5400 (3.8)	45	11.4	9.6	1.8
PVMOX-3.1k	3100 (2.17)	58	9.8	9.0	0.8
PVMOX-2.4k	2400 (2.9)	73	10.7	9.7	1.0
VMOX:MAP 1:1	2100 (1.39)	34–36	9.4	8.1	1.3
poly(VMOX:BuA) 9:1	2200 (2.83)	62	8.3	7.9	0.4
poly(VMOX:BuA) 8:2	2100 (2.15)	70	7.9	7.6	0.3
poly(VMOX:BuA) 7:3	2000 (1.75)	78	7.6	7.4	0.2
poly(VMOX:BuA) 6:4	1900 (1.60)		7.2	7.0	0.2
poly(VMOX:BuA) 5:5	1000 (1.76)		8.0	7.9	0.1

In the first study, a 1:1 VMOX:VCap copolymer did not perform better than PVMOX of similar molecular weight values.^21^ Therefore, we decided not to make other lactam copolymers, such as VP:VMOX copolymers, but to concentrate on other comonomers. We found that VMOX would not polymerize when mixed with NIPMAm using AIBN as an initiator; we only obtained a NIPMAm homopolymer.^21^ Somehow the VMOX was deactivated and did not take part in the polymerization. We thought hydrogen bonding from this group to VMOX may be deactivating the monomer causing it not to copolymerize. However, the related unmethylated monomer *N*-isopropylacrylamide (NIPAM) has been claimed to copolymerize with VMOX.^25^ We wondered if an acrylamide with tertiary amide group might fare better. We used the methacryloyl pyrrolidine (MAP) monomer, which has no N–H group. In addition, we deliberately chose the MAP monomer and not acryloylpyrrolidine (AP) since MAP does not homopolymerize under normal radical polymerization conditions, but AP does.^23^ This meant if MAP was to polymerize at all it had to copolymerize with VMOX. We polymerized VMOX and MAP in a 1:1 ratio with CTA in our standard iPrOH solvent. This time we did form a VMOX:MAP copolymer which gave a slightly opaque solution and a faint cloud point of about 34–36 °C at 2500 or 10,000 ppm in DI water. However, the molecular weight of VMOX:MAP was difficult to determine in pure DMF by GPC. It gave a broad peak with an average of 160 g/mol which would normally be indicative of no polymerization. However, the solution viscosity and cloud point of 34–36 °C indicated polymerization had taken place. Due to the sample having high polarity causing possible association with the column, it was reanalyzed using the addition of lithium chloride to the column solvent DMF. This gave an *M*_n_ value of 2100 g/mol (PDI = 1.39). The KHI performance of VMOX:MAP was fairly good with an average *T*_o_ of 9.4 °C and *T*_a_ value of 8.1 °C, suggesting that a polymer had indeed formed.

### Alkyl Acrylate:VMOX Copolymers and KHI Performance

Next, we investigated VMOX copolymers with non-amide vinylic monomers. There are a range of vinylic esters commercially available. We wanted to use an ester with about a C3–C4 hydrophobic group as these could also interact with the hydrate surfaces as well as increase the overall hydrophobicity of the copolymer relative to PVMOX.^26^ Alkyl acrylates and methacrylate ester monomers with C3–C4 groups are generally cheaper than vinyl alkanoate ester monomers. *n*-Butyl acrylate (BuA) is readily available and had been used previously for making KHIs based on VP:BuA copolymers, so we decided to use this monomer.^27−30^

We synthesized a range of VMOX:BuA copolymers with varying molar monomer ratios 9:1 down to 5:5. The molecular weights (*M*_w_) were in the range of 1000–2220 g/mol, suitable for optimal KHI performance for a monomodal distribution. Polymerization went to completion by ^1^H NMR spectroscopic analysis of the lack of vinyl protons. The copolymers with 7:3 or lower molar percentage of VMOX were hazy in solution at 2500 ppm with a small amount of deposit in the 5:5 ratio copolymer, which made cloud point evaluation difficult. This is probably due to the random nature of radical copolymerization giving some polymer strands with an even higher percentage of BuA which are too hydrophobic to be water-soluble. In fact, we were surprised that copolymers with such a high proportion of BuA to VMOX could be water-soluble and with high cloud points (62–78 °C). The relative polymerization rates of VMOX and BuA may be substantially different which may give polymer strands with some block characters. This may cause aggregation (micellization) to take place, giving better water solubility.

The KHI performance of the VMOX:BuA copolymers were all very similar giving average *T*_o_ values between 7.2 and 8.3 °C, the lowest being 7.2 °C for the 6:4 copolymer. This is significantly better than any of the PVMOX homopolymers in Table 2, indicating that the BuA monomer plays a role in improving the performance, as seen previously for VP:BuA copolymers. This was the first comonomer found to improve the KHI performance of VMOX polymers. The ability of the VMOX:BuA copolymers to arrest hydrate crystal growth was similar to that of the VMOX homopolymers as the *T*_o_ – *T*_a_ values were all low, maximum 0.4 °C.

As we had no VP:BuA copolymers available, we decided to make our own for comparison to VMOX:BuA copolymers. A 7:3 molar ratio VP:BuA copolymer was found to be insoluble in water. The polymerization rate of BuA is much greater than VP such that blocks of substantially the hydrophobic BuA monomer probably form. However, the 9:1 and 8:2 copolymers were water-soluble. The molecular weights from GPC determination indicated a bimodal distribution with a majority of low molecular weight. A 2500 ppm solution in DI water of the 8:2 copolymer had a cloud point at 40 °C, whereas the more hydrophilic 9:1 copolymer gave no cloud point. The 9:1 copolymer gave a poorer performance than PVP of similar molecular weight but the 8:2 copolymer has a lower average *T*_o_ value of 8.1 °C, similar to the value obtained for VMOX:BuA 8:2 copolymer. The crystal growth inhibition phase was similar for both copolymers. In summary, VMOX:BuA copolymers can give similar KHI performance as VP:BuA copolymers and with higher cloud points.

We also made a methacrylate:VMOX copolymer using THFMA. THFMA has a 5-ring tetrahydrofurfuryl pendant group and had been used previously to make good KHI polymers.^27,31^ THF is known to form sII hydrates so the tetrahydofurfuryl ring could give good interactions with open 5^12^6^4^ hydrate cavities on hydrate surfaces. We polymerized THFMA and VMOX in 1:1, 2:1, and 1:2 ratios, respectively. The first two ratio copolymers were insoluble in water, and the 1:2 was sparingly soluble even at 4 °C. Therefore, they were not tested for KHI performance.

### KHI Synergists

Having determined the performance of a range of low molecular weight VMOX homo- and co-polymers, we investigated if the KHI performance could be improved with solvents or chemicals known to be synergists for other KHI polymers. Table 3 summarizes the results. In most tests, we used a PVMOX sample with a molecular weight (*M*_w_) of 2400 g/mol. Using 2500 ppm polymer, we first added 5000 ppm of two amine oxides, tri(*n*-butyl)amine oxide (TBAO) and tri(iso-pentyl)amine oxide (TiPeAO). Both are known to be excellent synergists for VCap and NIPMAm polymers.^22,32^ In contrast, both amine oxides at 5000 ppm dosage were antagonistic to the performance of the PVMOX with TBAO being the worst. Considering these amine oxides are excellent THF hydrate crystal growth inhibitors, the results were surprising, although we have seen a strange effect where the performance of 2500+ ppm PNIPMAm is greatly reduced when mixed with 2500+ ppm TiPeAO.^33^ The same trend was also seen for tetra(*n*-pentyl)ammonium bromide (TPAB) another good crystal growth inhibitor. Addition of 5000 ppm TPAB to 2500 ppm PVMOX gave no significant change in the performance compared to the polymer by itself.

**Table 3:** Summary of SCC Rocking Cell Tests with 2500 ppm VMOX-Based Polymer plus Synergist

polymer	polymer concn., ppm	synergist	synergist conc., ppm	Av. *T*_o_, °C	Av. *T*_a_, °C	*T*_o_ – *T*_a_, °C
PVMOX-2.4k	2500			10.7	9.7	1.0
PVMOX-2.4k	5000			9.6	8.6	1.0
PVMOX-2.4k	2500	TBAO	5000	14.1	10.2	3.9
PVMOX-2.4k	2500	TiPeAO	5000	12.1	10.9	1.2
PVMOX-2.4k	2500	TPAB	5000	11.0	9.8	1.2
PVMOX-2.4k	2500	iBGE	5000	9.8	7.9	1.9
PVMOX-7.1k	2500	iBGE^a^	5000	8.2	7.6	0.8
PVMOX-2.4k	2500	iHexOl	5000	9.5	8.3	1.2
PVMOX-2.4k	2500	TMDD	2500	8.1	7.1	1.0
poly(VMOX-BuA) 6:4	2500			7.2	7.0	0.2
poly(VMOX:BuA) 6:4	2500	iBGE	5000	7.6	5.9	1.7
poly(VMOX:BuA) 6:4	2500	TMDD	2500	4.3	4.1	0.2
			1250	4.2	3.9	0.4

a PVMOX made as 33 wt % solution in iBGE as a solvent.

The rest of the potential synergists investigated were various types of alcohols and glycols. Like the amine oxides and TPAB, the alcohols and glycols also had alkyl groups of 4–6 carbon atoms, i.e., the correct size and shape for interacting with open cavities on gas hydrate surfaces. The isobutylated glycol ether, iBGE, is a useful high flash point synergist solvent for some KHI polymers.^27,34^ It was tested in two ways. First, VMOX monomer was polymerized in iBGE using an AIBN initiator to give PVMOX-7.1k with *M*_w_ 7100 g/mol as a 33 wt % in iBGE. KHI testing at 2500 ppm polymer meant 5000 ppm iBGE was also present. This gave an average *T*_o_ of 8.2 °C, 2.5 °C below the *T*_o_ value for PVMOX-2.4k (Table 2). In contrast, the addition of 5000 ppm iBGE to pre-made PVMOX-2.4k gave an average *T*_o_ value of 9.8 °C, only about 1 °C better than the polymer alone. Assuming the polymerization process is substantially the same for both polymers, this suggests that the PVMOX with higher molecular weight gave the best synergy with iBGE. This trend of synergist with polymer molecular weight has been seen previously for poly(*N*-isopropyl methacrylamide).^34^

4-Methyl-1-pentanol (iHexOl) had been shown previously to give excellent synergy with PVCap.^35^ With PVMOX the addition of 5000 ppm iHexOl lowered the average *T*_o_ value by 1.2 °C, a small but statistically significant improvement. The next candidate synergist was 2,4,7,9-tetramethyl-5-decyne-4,7-diol (TMDD), which has also been shown to have good synergy with PVCap, PNINPMAm and VP:VCap copolymer.^36^ A mixture of 2500 ppm TMDD with 2500 ppm PVMOX gave a *T*_o_ value of 8.1 °C, significantly better than either 2500 or 5000 ppm PVMOX.

Given the good synergetic performance of TMDD with PVMOX, we also investigated whether TMDD would also boost the performance of the poly(VMOX:BuA) 6:4 copolymer, which was the best copolymer tested. TMDD has limited water solubility, so we used a maximum 2500 ppm TMDD with 2500 ppm poly(VMOX:BuA) 6:4 copolymer. The average *T*_o_ value dropped from 7.2 to 4.3 °C. An almost identical value of 4.2 °C was obtained with the addition of 1250 ppm to the copolymer. This was the best synergetic effect and the lowest onset temperature measured in this whole study.

## Conclusions

KHIs are used to prevent deposits and plugging of oil and gas production flow lines by gas hydrates. The key ingredient in a KHI formulation is a water-soluble amphiphilic polymer. Recently, polymers of a new VMOX monomer were investigated as KHIs and shown to perform better than some commercial KHI polymers such as poly(*N*-vinyl pyrrolidone). This initial study has now been expanded to explore lower molecular weight PVMOX homopolymers and VMOX copolymers, both with improved KHI performance. VMOX:*n*-butyl acrylate copolymers in particular gave good KHI performance. In addition, a range of solvents and other small molecules, known to be good synergists for other KHI polymer classes, were investigated to find the most optimum combinations. The addition of 5000 ppm trialkylamine oxides (alkyl = butyl or iso-pentyl) and tetra(*n*-pentyl)ammonium bromide to 2500 ppm PVMOX was found to be antagonistic at the test concentrations while some alcohols and glycols were synergetic. The best synergist was 2,4,7,9-tetramethyl-5-decyne-4,7-diol. For example, a mixture of 2500 ppm with 2500 ppm PVMOX (*M*_w_ 2400 g/mol) performed significantly better than 5000 ppm PVMOX. We are continuing to investigate the KHI properties of VMOX-based polymers, including alternate test methods, the inhibition of structure I methane hydrate, performance in brines, and the presence of liquid hydrocarbons.
